# Gut microbiota and interstitial cystitis: exploring the gut-bladder axis through mendelian randomization, biological annotation and bulk RNA sequencing

**DOI:** 10.3389/fimmu.2024.1395580

**Published:** 2024-09-27

**Authors:** Chaowei Fu, Yu Zhao, Xiang Zhou, Jing Lv, Shengkai Jin, Yuhua Zhou, Fengping Liu, Ninghan Feng

**Affiliations:** ^1^ Jiangnan University Medical Center, Wuxi School of Medicine, Jiangnan University, Wuxi, Jiangsu, China; ^2^ Department of Urology, Jiangnan University Medical Center, Wuxi, Jiangsu, China

**Keywords:** Mendelian randomization study, interstitial cystitis, gut microbiota, gut-bladder axis, gene, causal relationship

## Abstract

**Background:**

Several observational studies have indicated an association between interstitial cystitis and the composition of the gut microbiota; however, the causality and underlying mechanisms remain unclear. Understanding the link between gut microbiota and interstitial cystitis could inform strategies for prevention and treatment.

**Methods:**

A two-sample Mendelian randomization analysis was conducted using published genome-wide association study summary statistics. We employed inverse variance weighted, weighted mode, MR-Egger, weighted median, simple mode, and cML-MA methods to investigate the causal relationship between gut microbiota and interstitial cystitis. Sensitivity analysis was performed to validate the results. Relevant gut microbiota was examined through reverse MR. Single nucleotide polymorphisms were annotated using FUMA to identify genes associated with these genetic variants, thereby revealing potential host gene-microbiota associations in interstitial cystitis patients.

**Results:**

Eight bacterial taxa were identified in our analysis as associated with interstitial cystitis. Among these, *Butyricimonas*, *Coprococcus*, *Lactobacillales*, *Lentisphaerae*, and *Bilophila wadsworthia* were positively correlated with interstitial cystitis risk, while taxa such as *Desulfovibrio piger*, *Oscillibacter unclassified* and *Ruminococcus lactaris* exhibited protective effects against interstitial cystitis. The robustness of these associations was confirmed through sensitivity analyses. Reverse MR analysis did not reveal evidence of reverse causality. Single nucleotide polymorphisms were annotated using FUMA and subjected to biological analysis. Seven hub genes (SPTBN1, PSME4, CHAC2, ERLEC1, ASB3, STAT5A, and STAT3) were identified as differentially expressed between interstitial cystitis patients and healthy individuals, representing potential therapeutic targets.

**Conclusion:**

Our two-sample Mendelian randomization study established a causal relationship between gut microbiota and interstitial cystitis. Furthermore, our identification of a host gene-microbiota association offers a new avenue for investigating the potential pathogenesis of interstitial cystitis and suggests avenues for the development of personalized treatment strategies.

## 1 Introduction

Interstitial cystitis (IC), also termed bladder pain syndrome (BPS), presents as a chronic non-bacterial inflammation of the bladder. It is characterized by varying degrees of frequency, urgency, dysuria, and/or suprapubic pain among affected individuals, sometimes extending to the urethra, perineum, vagina, and during sexual intercourse (1). The condition predominantly affects women, with a male-to-female ratio of approximately 1:10 (2) and an incidence rate as high as 6.53% (3) among adult women. Despite extensive research, the etiology of IC remains elusive (4), complicating the search for specific treatment modalities. Prolonged medical interventions for IC render patients vulnerable to mental health disorders such as depression and anxiety, contributing to a substantial suicide risk rate of 38.10% (5).

The human gut harbors a diverse microbiota comprising bacteria, fungi, archaea, viruses, and protozoa. This gut microbiota exists in symbiosis with the intestinal mucosa, playing crucial roles in immunomodulation, metabolism, and gastrointestinal protection in individuals with good health (6). Chronic dysbiosis can disrupt multiple bodily homeostatic mechanisms, potentially precipitating local and systemic inflammatory reactions (7). Researchers are increasingly intrigued by the interplay between gut microbiota and chronic inflammation-related disorders, as it unveils novel therapeutic avenues for conditions like inflammatory bowel disease (8). The gut microbiota has emerged as a critical factor in the pathogenesis of various inflammatory diseases, including asthma, inflammatory bowel disease, non-alcoholic fatty liver disease, rheumatoid arthritis, and type II diabetes mellitus (9). Recent studies have emphasized the “gut-bladder axis” in the interaction between the gut microbiota and bladder (10, 11). Similarly, research has explored the role of gut microbiota in the development and progression of IC and its implications for diagnostic and therapeutic strategies (12, 13). Comparisons with healthy controls revealed significant reductions in the levels of *Eggerthella sinensis*, *Collinsella aerofaciens*, *Faecalibacterium prausnitzii, Odoribacter splanchnicus, and Lactonifactor longoviformis*. An animal experiment demonstrated that fecal transplantation from wild-type mice into IC model mice alleviated IC symptoms, while fecal transplantation from IC model mice exacerbated pain and anxiety behaviors, suggesting the potential involvement of gut microbiota in IC development. However, comprehensive causal analysis of the interactions between gut microbiota and IC is still lacking in current literature.

Mendelian randomization (MR) is an innovative epidemiological method that utilizes genome-wide association study (GWAS) data and employs single nucleotide polymorphisms (SNPs) as instrumental variables (IVs) to establish causality and discern the causal relationship between an exposure and an outcome. The MR approach operates on the principle that distinct genotypes determine varied phenotypes, and adheres to Mendel’s Law of Inheritance, which stipulates that “parental alleles are randomly allocated to offspring during gamete formation.” Furthermore, alleles are randomly assigned to individuals at conception, predating both exposure and outcome, thereby obviating concerns of reverse causation. Theoretically, genetic variation remains unaffected by common confounders, such as postnatal environment and economic status. Notably, the MiBioGen consortium and the Dutch Microbiome Project (DMP) have recently published numerous loci linked to microbiome abundance, presenting an unparalleled opportunity to investigate causal relationships between gut microbiota and IC. Accordingly, we conducted a bidirectional two-sample MR analysis to elucidate the causal link between gut microbiota and IC while further probing the host gene-microbiota associations.

## 2 Materials and methods

**Figure 1:**
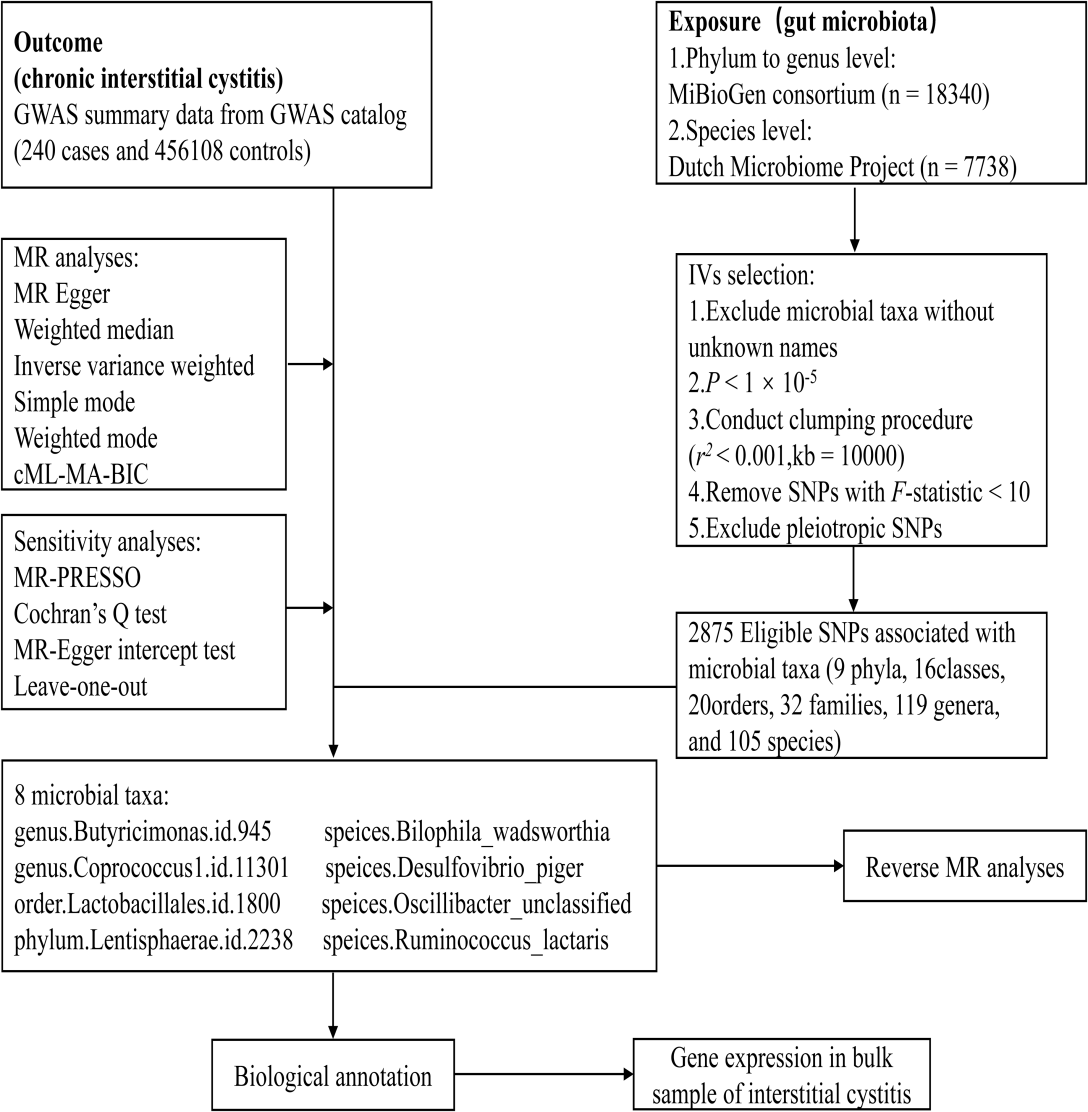
The flowchart of the study.

**Figure 1** presents an overview of the study. Since our study was based on a public database, no additional ethical approval or informed consent was required.

### 2.1 Data sources

Summary statistics of the human gut microbiome from phylum to genus level utilized in this research were acquired from the MiBioGen consortium. This consortium conducted a large-scale, multiethnic GWAS that integrated 16S ribosomal RNA gene sequencing and genotyping data from 18,340 participants across 24 cohorts spanning the United States, Canada, Israel, South Korea, Germany, Denmark, the Netherlands, Belgium, Sweden, Finland, and the United Kingdom. The aim was to investigate the relationship between autosomal human genetic variation and the gut microbiome. The dataset consisted of 211 taxa across 9 phyla, 16 classes, 20 orders, 35 families, and 131 genera. Notably, there were 12 unidentified genera and 3 unidentified families (14), with the genus being the lowest taxonomic level considered in this study. Furthermore, our investigation incorporated 105 additional species identified in a GWAS involving 7,738 individuals of European descent from the DMP. The composition of the gut microbiome was determined through shotgun metagenomic sequencing of stool samples as part of this initiative (15). Consequently, our study analyzed 9 phyla, 16 classes, 20 orders, 32 families, 119 genera, and 105 species-level taxa. Summary statistics for IC were extracted from the GWAS catalog website (16). The study utilized the phenotype “chronic interstitial cystitis” with the GWAS summary data encompassing 456,348 European adult female participants, comprising 240 cases and 456,108 controls. Additional details regarding the GWAS conducted in this Mendelian randomization study are available in **Supplementary Table S1**.

### 2.2 MR

The usage and interpretation of our MR study adhere to the STROBE-MR (Strengthening the Reporting of Observational Studies in Epidemiology-Mendelian Randomization) checklist (17, 18) (**Supplementary Table S2**).

The MR analysis relies on three key assumptions: 1) the IVs are strongly associated with the exposure, 2) the IVs are not associated with confounding factors between exposure and outcome, and 3) the IVs influence outcomes solely through the exposure. In summary, the gut microbiota was considered the exposure, with IC serving as the outcome. Bacterial taxa were examined at six levels (phylum, class, order, family, genus, and species), and a unique taxon was defined as a characteristic. To ensure the reliability and accuracy of the conclusions regarding the causal relationship between gut microbiota and IC, the following quality control measures were implemented to select IVs. The selection criteria were as follows (19, 20): 1) Criteria were relaxed appropriately due to the limited number of IVs at *P* < 5 × 10^-8^. SNPs associated with each taxon were chosen as potential IVs at the genome-wide significance threshold (*P* < 1 × 10^-5^); 2) Using data from 1,000 samples from the European Genome Initiative as a reference panel, the linkage disequilibrium (LD) between these SNPs was calculated, and SNPs meeting an *r^2^* < 0.001 (clumping window size = 10,000kb) were selected to reduce the likelihood of biased results; 3) SNPs with minor allele frequencies (MAF) ≤ 0.01 were excluded; and (4) An essential step in MR is to ensure that the effect of SNPs on exposure corresponds to the same allele as the effect on the outcome. To mitigate distortions in strand orientation or allele coding, palindromic SNPs were excluded. Additionally, for each SNP, the strength of the IVs was evaluated using the F-statistic, calculated by the formula F=(R^2^× (N-2))/(1- R^2^), and R^2^=(2 × β^2^ × EAF × (1-EAF))/(2 ×β^2^ × EAF × (1-EAF) + 2 × SE^2^ × N × EAF × (1-EAF)) with R^2^ representing the proportion of variance in exposure explained by the genetic variables, N indicating the number of participants, EAF denoting the effect allele frequency, and β representing the estimated effect of SNP to assess its ability to predict the outcome uniquely (21–23). A significant weak instrumental bias was deemed absent if the corresponding F-statistic exceeded 10 (24).

### 2.3 Reverse MR

To evaluate the causal relationship between gut microbiota and IC, we conducted reverse MR analysis using the same selection criteria for IVs and MR analysis methodology as described above. This analysis was performed on the SNPs associated with IC to investigate whether IC exerts a causal influence on gut microbiota. The data sources used for reverse Mendelian randomization were identical to those utilized for forward Mendelian randomization.

### 2.4 Statistical analysis

MR analysis was conducted to explore the causal relationship between microbiota features and IC. The Wald ratio test estimated the association between identified IVs and IC for features with only one IV (25). For features with multiple IVs, five widely used MR methods were employed: inverse variance weighted (IVW) (26), weighted mode (27), MR-Egger regression (28), weighted median (29), and simple mode (27). Additionally, this study utilized a model based on the Constrained Maximum Likelihood and Model Averaging MR method (cML-MA) (30), which does not depend on internal assumptions and is used to account for correlated and uncorrelated multivariate effects. IVW was used as the primary MR Analysis method to assess the relationship between gut microbiota and IC, supplemented by the other five methods. Estimates were presented as odds ratios (ORs) with 95% confidence intervals (CIs).

Sensitivity analyses were conducted to assess the robustness of the results, including heterogeneity tests, pleiotropy tests, and leave-one-out sensitivity tests. The pleiotropy test comprised MR-Egger intercept and MR-pleiotropy residual sum and outlier (MR-PRESSO) tests. A significant intercept term (*P* ≤ 0.05) in the MR-Egger analysis indicated potential pleiotropy. Horizontal pleiotropy was identified using MR-PRESSO, and any outliers detected prompted their removal, followed by reanalysis of the remaining IVs (31). Cochran’s Q test determined the heterogeneity of single nucleotide polymorphisms (SNPs), with a statistically significant Cochran’s Q test (*P* ≤ 0.05) indicating significant heterogeneity in the analysis results (32, 33). Leave-one-out sensitivity tests were conducted to ascertain whether a single SNP was driving the causal signal. This method compared differences in exposure and outcome of IVs. If the IVs explained more of the difference in exposure than in outcome, the identified causal link could be considered directionally plausible (34).

Additionally, we applied the Bonferroni method at each taxonomic level (phylum, order, family, genus, and species) to determine distinct significance thresholds for multiple testing based on the number of bacteria at each level. The significance threshold was defined as *P* ≤ 0.05/n, where n represents the effective number of independent bacterial taxa at the corresponding level. For example, at the phylum level, the threshold was 5.6 × 10^-3^ (0.05/9), at the class level, it was 3.1 × 10^-3^ (0.05/16), at the order level, it was 2.5 × 10^-3^ (0.05/20), at the family level, it was 1.6 × 10^-3^ (0.05/32), at the genus level, it was 4.2 × 10^-4^ (0.05/119), and at the species level, it was 4.8 × 10^-4^ (0.05/105). Bacterial taxa with *P* ≤ 0.05, although not statistically significant after Bonferroni correction, were considered potentially associated, and corrected *P*-values that remain significant are considered highly likely to be associated with IC.

The analysis was conducted using the “TwoSampleMR (version 0.5.7) (34)”, “MRcML (30)”, and “MRPRESSO (version 1.0) (31)” packages within the R program (version 4.3.1).

### 2.5 Biological annotation

To delve deeper into the mechanism of gut microbiota’s influence on IC, we identified IVs that passed the screening criteria in the MR analysis for each taxon as lead SNPs in FUMA GWAS (35), a comprehensive platform for annotating, prioritizing, visualizing, and interpreting GWAS results. Using the SNP2GENE tool in FUMA, we localized these SNPs to genes (36). Independent significant SNPs aid in identifying multiple genetic signals associated with traits or diseases, ensuring signal independence and preventing redundancy due to linkage disequilibrium. Lead SNPs are further refined to select the most representative variants, which are then used to define genomic risk loci and for subsequent functional studies or further analysis as key genetic markers. Independent significant SNPs were defined with a *P* < 1×0^-5^ and pairwise LD (*r^2^*) < 0.6, whereas lead SNPs were identified with a stricter threshold of *r^2^* < 0.1. Closely located independent significant SNPs (< 250kb based on the most right and left SNPs from each LD block) formed a genomic risk locus. We employed three strategies—position mapping (with default 10kb window), expression quantitative trait loci (eQTL) mapping (tissue type for eQTLGen cis-eQTLs and eQTLGen trans-eQTLs), and 3D chromatin interaction mapping (builtin chromatin interaction data for bladder)—to annotate SNPs to genes and generate a set of genes. To explore gene interactions at the protein level, we constructed a protein-protein interaction (PPI) network of mapped genes using STRING (37), with 0.4 serving as the recommended minimum interaction index and all other variables set to default values. We conducted Gene Ontology (GO) enrichment analysis of these genes using KOBAS (38) to understand the molecular mechanisms associated with the potential relationship between gut microbiota influence and IC. The significance threshold was set at *P* ≤ 0.05. Finally, we utilized the Cytoscape plugin “CytoHubba” to identify hub genes within the PPI network. We applied the Maximal Clique Centrality (MCC) method to pinpoint the top five hub genes, as gene nodes with numerous interactions are pivotal for maintaining the stability of the entire network (39).

### 2.6 Bulk RNA data analysis

We obtained data GSE11783 from the Gene Expression Omnibus (GEO) database (40). This dataset presents reliable gene expression profiles of IC bladder tissues exclusively from human samples, comprising 10 IC patient bladder samples and 6 healthy control bladder samples. The dataset was generated using the GPL570[HG-U133_Plus_2] Affymetrix Human Genome U133 Plus 2.0 Array platform. After standardizing, normalizing, annotating, and cleaning the clinical information within the GSE11783 dataset, we identified differentially expressed genes (DEGs) between the IC patient group and the healthy control group using GEO’s GEO2R tool. The criterion for identifying DEGs was set at *P* ≤ 0.05.

## 3 Results

### 3.1 Instrumental variables selection

We utilized 2875 SNPs as IVs for the gut microbiota according to the IV selection criteria. Effect alleles, alternative alleles, β coefficients, standard errors (SE), and P-values of these SNPs were extracted for MR analyses. The F-statistics of the IVs were all >10, indicating no evidence of weak instrumental bias. **Supplementary Table S3** provides detailed information on the selected IVs.

### 3.2 Mendelian randomization analysis

**Figure 2:**
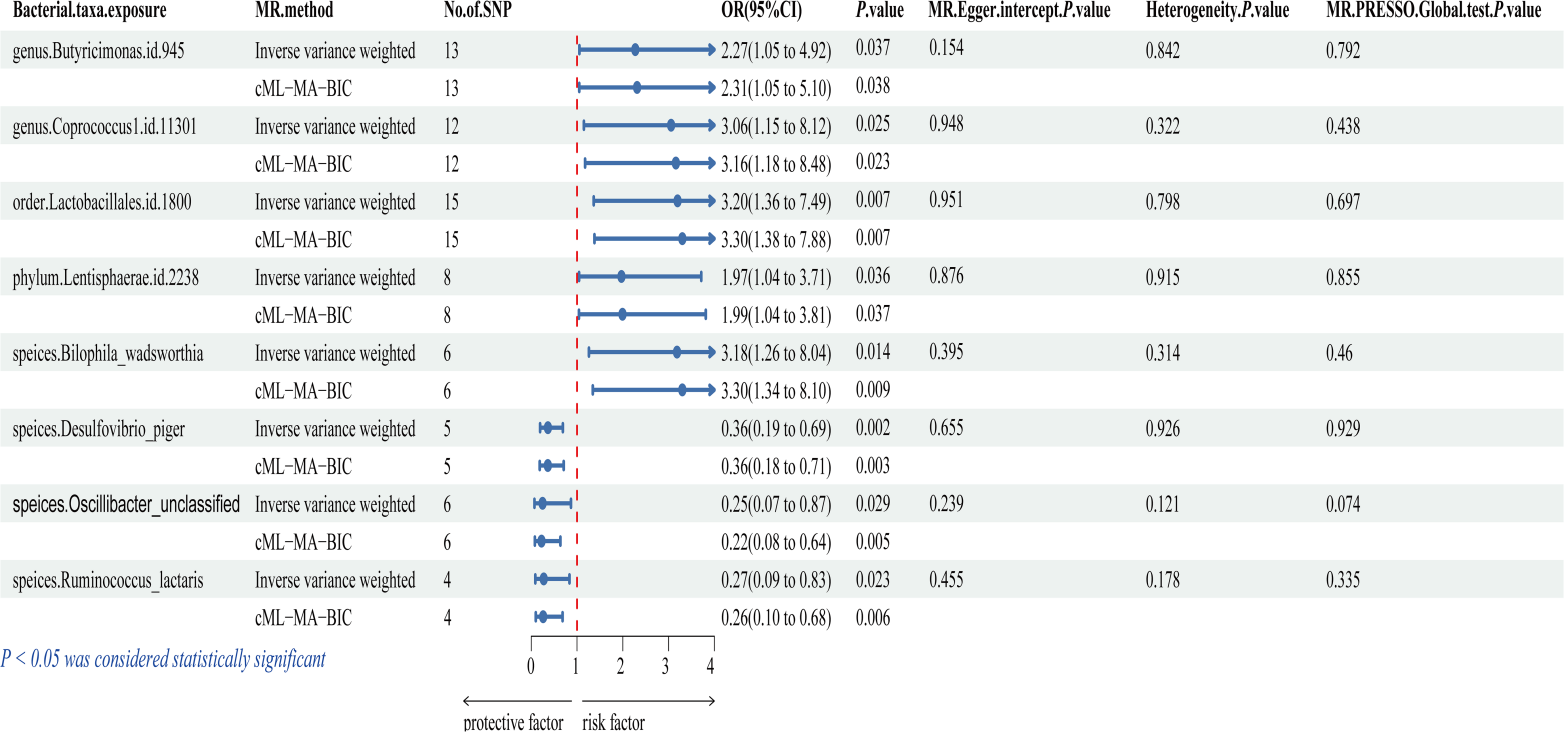
Forest plot of the associations between genetically determined 8 gut microbial genera with the risks of IC. OR, odds ratio; CI, confidence interval; SNP, single nucleotide polymorphism.

**Figure 2** and **Supplementary Table S4** illustrate that eight bacterial taxa are associated with IC, namely *genus.Butyricimonas.id.945*, *genus.Coprococcus1.id.11301*, *order.Lactobacillales.id.1800*, *phylum.Lentisphaerae.id.2238*, *speices.Bilophila_wadsworthia*, *speices.Desulfovibrio_piger*, *speices.Oscillibacter_unclassified*, and *speices.Ruminococcus_lactaris*. The cML-MA, MR-Egger regression, weighted mode, simple mode, and weighted median methods provided comparable causal estimates to IVW in terms of magnitude and direction (**Supplementary Figure S1**).

The study revealed a positive association between IC risk and five gut microbiota: *genus.Butyricimonas.id.945* (OR = 2.273, 95% CI: 1.052-4.915, *P* = 0.037), *genus.Coprococcus1.id.11301* (OR = 3.055, 95% CI: 1.150-8.117, *P* = 0.025), *order.Lactobacillales.id.1800* (OR = 3.196, 95% CI: 1.365-7.485, *P* = 0.007), *phylum.Lentisphaerae.id.2238* (OR = 1.970, 95% CI: 1.045-3.714, *P* = 0.036), and *speices.Bilophila_wadsworthia* (OR = 3.184, 95% CI: 1.260-8.043, *P* = 0.014). These findings suggest a potential increase in IC risk associated with these bacteria. Conversely, three gut microbiota were linked to a reduced risk of IC: *speices.Desulfovibrio_piger* (OR = 0.364, 95% CI: 0.193-0.690, *P* = 0.002), *speices.Oscillibacter_unclassified* (OR = 0.247, 95% CI: 0.070-0.868, *P* = 0.029), and *speices.Ruminococcus_lactaris* (OR = 0.273, 95% CI: 0.089-0.835, *P* = 0.023). These findings suggest a potential protective role against IC. However, after applying multiple comparison correction, these associations are no longer significant. We recognize the importance of the Bonferroni correction in controlling for multiple comparisons. Nonetheless, Bonferroni correction is highly conservative and can lead to over-correction, potentially obscuring biologically meaningful signals when dealing with a large number of comparisons. This is particularly relevant in studies of complex traits, where certain small yet biologically significant effects may not remain significant after rigorous correction. Although the associations are no longer significant post-correction, the *P*-values still suggest potential biological mechanisms (41, 42) and may indicate a link between specific gut microbiota and IC. We propose that these preliminary findings serve as important clues for future research and can provide valuable insights for developing new hypotheses and experimental designs. Therefore, we highlight the significance of these results and advocate for further investigation into their potential biological relevance.

### 3.3 Sensitivity analysis

The MR-Egger intercept analysis yielded non-significant results (*P* > 0.05), suggesting no genetic pleiotropy bias in the results. MR-PRESSO analysis also detected no outliers (*P* > 0.05), and Cochran’s Q test found no significant heterogeneity (*P* > 0.05). Collectively, these sensitivity analyses, including Cochran’s Q test, MR-Egger intercept, MR-PRESSO global test, and leave-one-out test, underscored the robustness of the MR results across both samples (**Supplementary Tables S5**-**S7**). Leave-one-out analysis revealed that the majority of correlated signals were not driven by a single genetic marker (**Supplementary Figure S2**). Additionally, funnel plots demonstrating a symmetrical pattern were provided to affirm the reliability of the results (**Supplementary Figure S3**).

### 3.4 Reverse MR analysis

To investigate potential reverse causal effects, we examined IC as the exposure and gut microbiota as the outcome. Employing the same IV selection criteria as the forward MR analysis, detailed IV information is provided in **Supplementary Table S8**. Subsequently, we conducted a reverse MR analysis using identical methodology to explore the causal relationship between the eight bacterial taxa and IC.

Results depicted in **Supplementary Table S9** showed that none of the gut microbiota exhibited a significant inverse causal relationship with IC, including *genus.Butyricimonas.id.945* (*P* = 0.381), *genus.Coprococcus1.id.11301* (*P* = 0.183), order.Lactobacillales.id.1800 (*P* = 0.593), *phylum.Lentisphaerae.id.2238* (*P* = 0.646), *speices. Bilophila_wadsworthia* (*P* = 0.736), *speices.Desulfovibrio_piger* (*P* = 0.894), speices. Oscillibacter_unclassified (*P* = 0.168), and *speices.Ruminococcus_lactaris* (*P* = 0.486). Cochran’s Q test indicated no significant heterogeneity in IVs. Additionally, results from MR-Egger intercept analysis and MR-PRESSO analysis did not reveal significant horizontal pleiotropy (**Supplementary Tables S10**-**S12**).

### 3.5 Biological annotation

**Figure 3:**
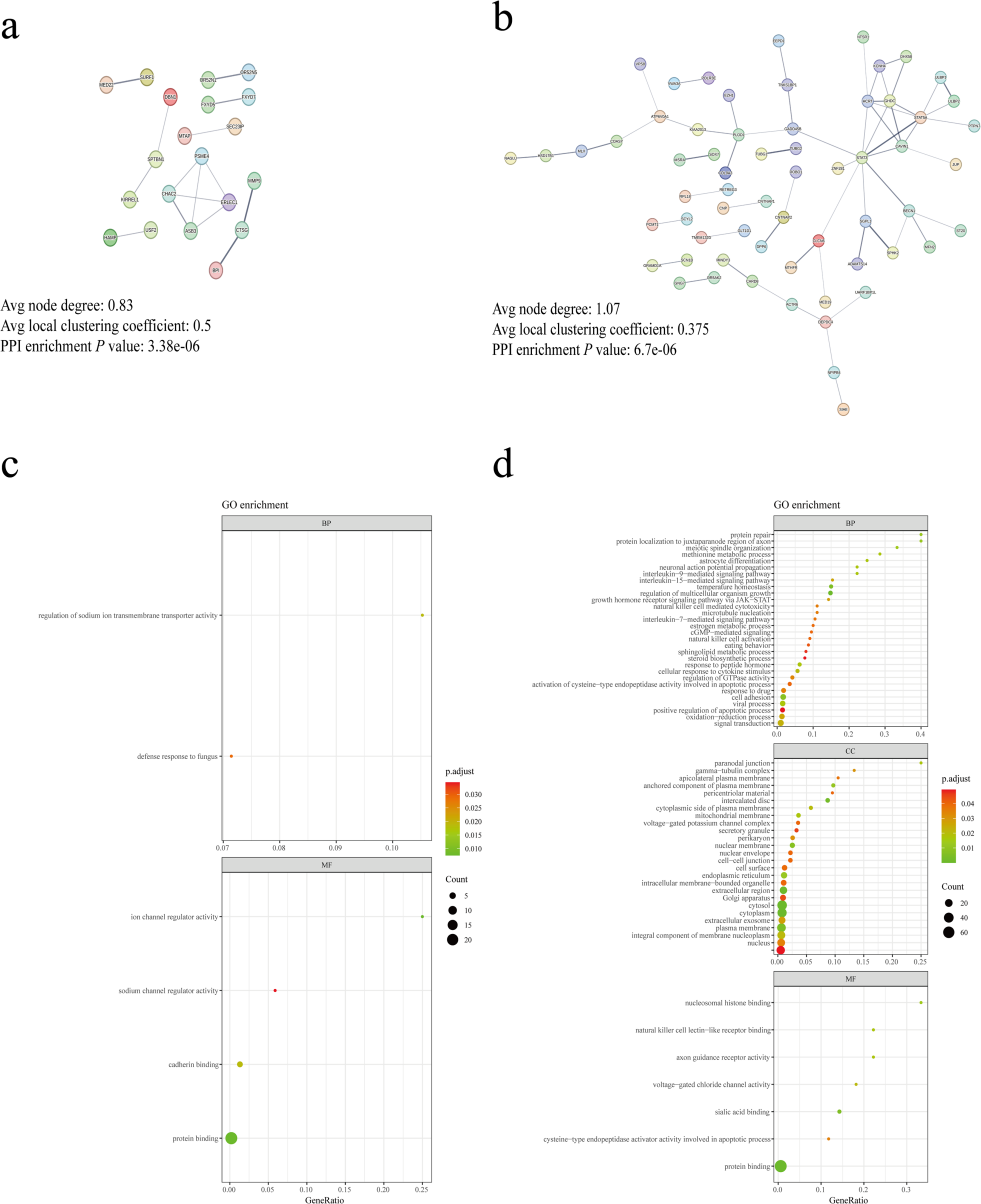
Biological annotation of genes. **(A)** PPI network of genes associated with gut microbiota linked to decreased IC risk. **(B)** PPI network of genes associated with gut microbiota linked to increased IC risk. Edge colors represent different evidence underlying predicted protein–protein interactions in the STRING database. Line thickness indicates the strength of data support and disconnected nodes are hidden in the network. **(C)** Enrichment analysis of GO terms for genes associated with gut microbiota linked to decreased IC risk. **(D)** Enrichment analysis of GO terms for genes associated with gut microbiota linked to increased IC risk. BP, biological process; CC, cellular component; MF, molecular.

Initially, we employed the SNP2GENE tool in FUMA to target SNPs associated with bacterial taxa influencing increased and decreased IC risk, resulting in the identification of 116 and 38 genes, respectively (**Supplementary Table S13**). To elucidate gene interactions at the protein level, we utilized STRING to construct PPI networks (**Figures 3A, B**), illustrating the first 5 nodes as hub genes. Notably, hub genes for bacterial taxa associated with increased IC risk included STAT3, GHDC, HCRT, STAT5A, and CAVIN1, while those linked with decreased IC risk comprised ASB3, ERLEC1, CHAC2, PSME4, and SPTBN1. Furthermore, we performed GO enrichment analysis on the genes mapped through the aforementioned methods, categorizing them into molecular function, cellular composition, and biological process. Our findings revealed that bacterial taxa elevating IC risk (**Figure 3C**) predominantly localized within the cytosol, cytoplasm, plasma membrane, and nucleus. Moreover, they were primarily associated with protein binding at the molecular level, while being involved in signal transduction and cell adhesion processes at the biological level. Conversely, bacterial taxa mitigating IC risk (**Figure 3D**) exhibited molecular functions primarily related to protein binding, cadherin binding, and channel regulator activity. Their biological processes primarily pertained to the regulation of transmembrane transporter activity.

### 3.6 Bulk RNA analysis

**Figure 4:**
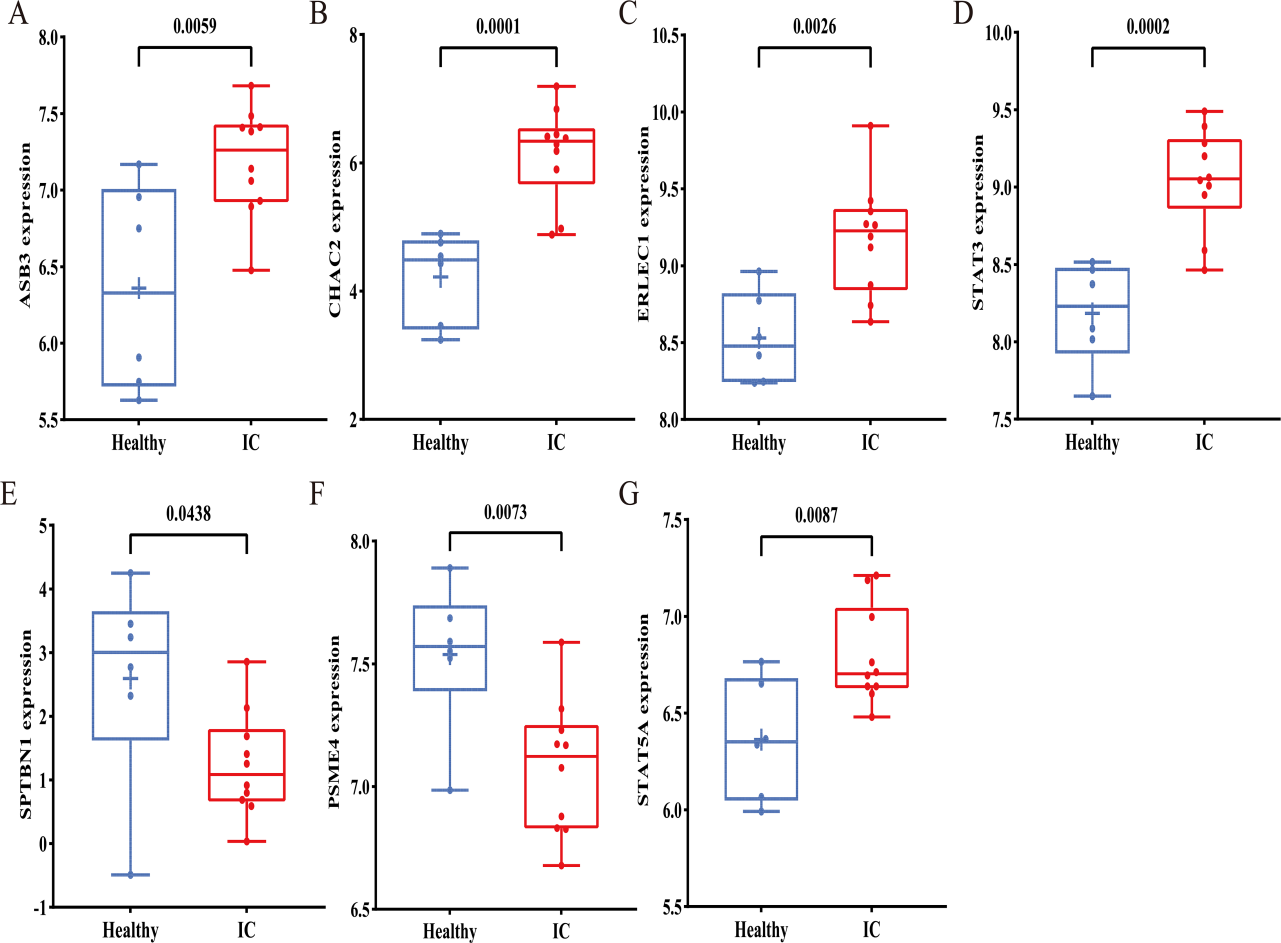
Gene expression in healthy people and IC patients. Expression of ASB3**(A)**, CHAC2 **(B)**, ERLEC1 **(C)**, STAT3 **(D)**, SPTBN1 **(E)**, PSME4 **(F)**, and STAT5A **(G)** in healthy people and IC patients.

Using the GSE11783 dataset, the normalized results are depicted in **Figure S4**. We conducted differential gene expression analysis between healthy individuals and IC patients using GEO2R. Our findings revealed differential expression in 7 out of 10 central genes compared to healthy individuals. Notably, SPTBN1 and PSME4 exhibited significant down-regulation in IC patients, while CHAC2, ERLEC1, ASB3, STAT5A, and STAT3 showed significant up-regulation in IC patients (**Figure 4**). Therefore, these differentially expressed genes (DEGs) may play pivotal roles in IC development influenced by gut microbiota.

## 4 Discussion

The investigation initiated with MR analysis of two samples to explore the potential causal link between gut microbiota and IC, employing summary statistics from GWAS. Subsequently, we delved into the molecular mechanisms through which gut microbiota influences IC development and elucidated potential host gene-microbiota associations in IC patients. To our knowledge, this represents the inaugural MR study probing the causal association between gut microbiota and IC. Our study not only holds promise for effective IC prevention and treatment strategies but also furnishes novel insights into IC pathogenesis from a gut microbiota perspective.

In our present MR investigation, we identified a causal link between eight bacterial taxa and the risk of IC. Notably, *Butyricimonas*, *Coprococcus*, *Lactobacillales*, *Lentisphaerae*, and *Bilophila wadsworthia* exhibited positive associations with IC risk. Conversely, taxa like *Desulfovibrio piger*, *Oscillibacter*, and *Ruminococcus lactaris* demonstrated protective effects against IC.

*Desulfovibrio piger*, one of the most prevalent pairs of sulfate-reducing bacteria in feces (43), releases hydrogen sulfide (H2S), a third gas signaling molecule widely involved in various biochemical reactions in the animal body. It plays a physiological role in the gastrointestinal tract, regulating cellular and tissue functions (44, 45). It can downregulate the expression of cyclooxygenase-2 mRNA and prostaglandin synthesis, as well as reduce the expression of the pro-inflammatory cytokine tumor necrosis factor-alpha mRNA, thereby exerting either pro-inflammatory or anti-inflammatory effects. Its anti-inflammatory effects may be one of the mechanisms through which it benefits individuals with IC (46, 47). Additionally, H₂S may also function as a signaling molecule in the host, potentially providing beneficial effects such as cardioprotection (48). Recent research has revealed *Desulfovibrio piger*’s positive impact on the growth and function of *Faecalibacterium prausnitzii* (49). *Faecalibacterium prausnitzii*, a crucial bacterium in the human gut flora, is a major producer of butyric acid, known for its anti-inflammatory effects, enzyme activity maintenance, and protection of the digestive system against intestinal pathogens (50). However, observational studies indicate significantly reduced levels of *Faecalibacterium prausnitzii* in the feces of IC patients (13). *Ruminococcus*, one of the earliest gastric bacteria identified, significantly influences metabolism, digestion, and the metabolism of resistant starch. Patients with inflammatory bowel disease often exhibit varied *Ruminococcus* numbers and activities in their intestines, affecting gut health and inflammation levels (51). Anomalies in *Ruminococcus* may contribute to immune-related diseases like allergy, eczema, and asthma (52). Although the precise mechanism remains unclear, studies suggest that *Ruminococcus* may impact the pathogenesis of immune-mediated diseases by influencing intestinal immune system regulation and inflammatory responses. *Bilophila*, an opportunistic pathogen group, represented by *Bilophila wadsworthia*, was initially isolated from patients with gangrenous and perforated appendicitis (53). It synergizes with a high-fat diet, leading to increased inflammatory responses, intestinal barrier dysfunction, and bile acid metabolism abnormalities, consequently raising the risk of glucose metabolism abnormalities and hepatic steatosis (54). *Bilophila wadsworthia* eventually metabolizes 2,3-dihydroxypropane-1-sulfonate, producing H2S through a recently discovered metabolic pathway (55). *Butyricimonas*, a beneficial bacterium, produces short chain fatty acids (SCFAs), the primary metabolites of anaerobic bacterial fermentation in the gut (56). SCFAs serve as crucial fuel for intestinal epithelial cells and exhibit potential anti-inflammatory effects, along with immune cell modulation (57). For example, SCFAs influence epithelial barrier function and mucosal and systemic immunity through G protein-coupled receptor signaling or histone deacetylase activity. SCFAs can also directly or indirectly affect immune responses in extramural sites such as the liver, lungs, reproductive tract, and brain, and are associated with various diseases. *Coprococcus*, a significant member of the *Firmicutes* phylum and *Lachnospiraceae* family, resembles *Faecalibacterium prausnitzii* in terms of its beneficial role in intestinal health and immune system balance. Actively fermenting carbohydrates, *Coprococcus* is a key butyric acid producer and serves as a microbial biomarker for assessing gastrointestinal health (58). Butyric acid exerts anti-inflammatory effects by directly influencing the differentiation of intestinal epithelial cells, phagocytes, B cells, plasma cells, as well as regulatory and effector T cells (57). Interestingly, we observed deleterious effects of the *Butyricimonas* and *Coprococcus* on IC, offering a novel perspective for future investigations.

Studies have demonstrated that gut microbiota can regulate host physiology and potentially induce disease by influencing gene expression (59–61). Thus, we utilized FUMA to map SNPs to genes and explore genes that might exhibit a causal relationship with IC. To gain insight into the biological functions of these genes in disease, we conducted GO enrichment analysis. Our findings suggest that gut microbiota with beneficial roles may impact channel regulator activity, protein binding, cadherin binding, and other pathways. Conversely, gut microbiota with detrimental roles may influence protein repair, paranodal junction, cell-cell junction, protein binding, and other pathways. Subsequently, we conducted a comparative analysis of hub gene expression between IC patients and healthy controls using bulk RNA analysis. The results revealed significant down-regulation of SPTBN1 and PSME4 in IC patients, while CHAC2, ERLEC1, ASB3, STAT5A, and STAT3 were significantly up-regulated. Therefore, SPTBN1, PSME4, CHAC2, ERLEC1, ASB3, STAT5A, and STAT3 could potentially represent novel genes implicated in the underlying pathogenesis of IC and serve as therapeutic targets.

Spectrin Beta, Non-Erythrocytic 1 (SPTBN1) is a protein-coding gene known for its role in maintaining neuronal morphological stability, cellular structure, and cell signaling. Additionally, it contributes to synapse formation and normal neuronal function (62). Several studies have indicated a correlation between IC and neurological disorders such as fibromyalgia, depression, and memory impairment (63, 64). Moreover, neurogenic pain is considered a potential etiological factor in IC, wherein pain may arise from dysfunction in peripheral, central, and/or endogenous inhibitory pathways (65). Proteasome Activator Subunit 4 (PSME4) encodes a protein that regulates proteasome activity, thereby influencing the rate and selectivity of protein degradation (66). However, its association with inflammation and IC remains unclear. One of the primary intracellular functions of the protein encoded by the ChaC Glutathione Specific Gamma-Glutamylcyclotransferase 2 (CHAC2) gene, is its involvement in glutathione metabolism. Glutathione, a crucial antioxidant, safeguards cells from oxidative stress (OS)-induced damage within the cell. Research indicates that the expression level of the CHAC2 protein is influenced by intracellular OS levels, thereby regulating intracellular glutathione levels and subsequently impacting the cell’s redox state and its ability to counter OS (67, 68). OS is recognized as a significant contributing factor in inflammatory diseases. Notably, one of the key features underlying the pathobiology of IC is the excessive production of reactive oxygen species and cytokines, leading to pronounced OS (69). Mitigating OS has emerged as a potential therapeutic approach for IC (70, 71). The Endoplasmic Reticulum Lectin 1(ERLEC1) gene encodes a protein pivotal in the endoplasmic reticulum, particularly in protein quality control and folding. It aids in identifying proteins folding abnormally or with specific sugar groups, thereby participating in their subsequent processing and modification, including their targeting to appropriate pathways for degradation or repair (72). However, its association with inflammation and IC remains unclear. Ankyrin Repeat and SOCS Box Containing 3 (ASB3) is a protein-coding gene containing the SOCS box, facilitating the binding and degradation of various proteins. Members of the SOCS protein family are known to impede cytokine signaling by directly interacting with JAK kinase or activated cytokine receptors (73). Although ASB3 genes have been extensively studied in oncological disorders, such as promoting colorectal cancer cell growth and metastasis when mutated or down-regulated (74), their connection with inflammation and IC remains unelucidated. Signal Transducer and Activator of Transcription 5A (STAT5A) and Signal Transducer and Activator of Transcription 3 (STAT3) are pivotal transcription factors involved in various biological processes. Both are integral components of the Janus kinase/signal transducer and activator of transcription (JAK/STAT) signaling pathway, which plays a critical role in regulating cell growth, differentiation, survival, and immune response (75). Moreover, the JAK/STAT pathway modulates the production of numerous inflammatory mediators, thereby regulating inflammation development (76). There is now a degree of certainty that inflammation plays a key role in the pathophysiology of IC (77). A prior investigation observed significant enrichment of the JAK/STAT signaling pathway in a cyclophosphamide (CYP)-induced IC mouse model (78), where pSTAT3 expression increased post-CYP treatment in a rat cystitis model (79). Inhibition of the JAK/STAT signaling pathway demonstrated effectiveness in reducing CYP-induced inflammation in rat ulcerative cystitis (80). Hence, we hypothesize that gut microbiota may contribute to the onset and progression of IC inflammation by modulating the JAK/STAT signaling pathway, thereby influencing IC inflammation development. In conclusion, further studies are warranted to elucidate the mechanisms underlying the action of these genes in IC and their therapeutic potential.

IC, an autoimmune disease, is characterized by the infiltration of immune cells such as lymphocytes, macrophages, and mast cells into the bladder tissue of affected individuals. The activation and proliferation of these immune cells can lead to inflammation and damage to the bladder mucosa, resulting in symptoms such as pain, frequent urination, and urgency (81). Previous studies have shown that the development of IC involves the infiltration of various immune cells, including T cells, B cells, plasma cells, macrophages, neutrophils, and mast cells (81, 82). Growing evidence suggests that the gut microbiota plays a crucial role in shaping the immune system. First, the gut microbiota influences the systemic immune response by regulating intestinal barrier function. A healthy microbiota maintains the integrity of the intestinal barrier, preventing the invasion of harmful substances and pathogens, thereby reducing systemic inflammation. Conversely, an imbalanced gut microbiota can lead to barrier disruption, loss of immune tolerance, and chronic inflammation, which can affect autoimmune responses (83). Second, the gut microbiota directly impacts immune cell function through the production of various metabolites, such as SCFAs. For example, SCFAs produced from the metabolism of dietary fibers stimulate oxidative phosphorylation and glycolytic activity in CD8^+^ T cells, enhancing their effector functions. SCFAs also activate glycolysis in B cells via mTOR signaling (84). These metabolites can influence the immune cells in the bladder through the bloodstream. Moreover, a key role of the gut microbiota in the immune system is the promotion of immune tolerance. The immune system must recognize and attack pathogenic microbes while tolerating the normal microbiota. The gut microbiota assists in distinguishing harmful microbes from benign ones by producing signaling molecules and regulating immune cell functions. This helps prevent autoimmune diseases, although dysbiosis can disrupt immune tolerance and trigger autoimmune reactions (85). Looking ahead, integrating GWAS summary statistics on interstitial cystitis with single-cell RNA sequencing data (86), or employing new analytical methods, such as ‘scPagwas’ (87), could provide deeper insights into the immune cell heterogeneity in the bladder tissue of IC patients and elucidate the specific roles of these cells in IC pathogenesis. In summary, the interaction between the gut microbiota and the immune system is pivotal in the pathogenesis of IC. Understanding how the gut microbiota affects immune cells and their roles in IC could offer new insights for the diagnosis and treatment of this condition.

This study has several strengths. By employing MR analysis, we established a causal relationship between gut microbiota and IC, thereby controlling for confounding factors and reverse causation. Additionally, we utilized the cML-MA method to address biases due to correlated and uncorrelated pleiotropy and applied a two-sample MR design with non-overlapping exposure and outcome summary data to mitigate bias. While most current research focuses on analyses at the genus level, our study is the first to identify bacteria closely related to IC at the species level, specifically *Desulfovibrio piger*, *Oscillibacter unclassified*, and *Ruminococcus lactaris*. Identifying these species provides new insights into the gut microbiota’s role in IC. Future research should explore the potential of regulating these key bacterial taxa through fecal microbiota transplantation or probiotic treatments to alleviate IC symptoms. Increasing the abundance of *Desulfovibrio piger*, *Oscillibacter unclassified*, and *Ruminococcus lactaris* could offer effective intervention targets. For instance, enhancing the abundance of *Desulfovibrio piger* has been shown to positively influence the growth and function of the gut probiotic *Faecalibacterium prausnitzii*, suggesting new possibilities for personalized IC treatment. These new findings not only enhance our understanding of the etiology of IC but also provide clear directions for developing new clinical treatment strategies, particularly through microbiota modulation for symptom management. Such strategies may involve targeted supplementation or suppression of specific bacterial species, offering new therapeutic hope for IC patients. Additionally, we analyzed the associations between host genes and the microbiota, further exploring potential therapeutic targets and mechanisms underlying IC pathogenesis.

However, our study has several limitations. Firstly, the majority of patients in the GWAS summary data used were of European descent, with limited representation from other ethnicities. This imbalance may introduce estimation bias and limit the generalizability of our findings. Consequently, the results may not be fully applicable to individuals of non-European origin. Future MR studies exploring the causal relationship between gut microbiota and IC should include diverse European and non-European populations to enhance generalizability. Secondly, subgroup analyses, such as with Hunner-type and non-Hunner-type, were not possible since summary data, rather than raw data, were used in the analyses, whereas symptoms are usually more severe in the former (88). Moreover, IC predominantly affects the female population, and gender-specific differences exist in gut microbiota composition (89). Unfortunately, our study did not conduct separate analyses for both genders. Future investigations should incorporate gender-specific MR analysis to provide comprehensive insights into the relationship between gut microbiota and IC across different populations. Then, although relaxing the *P*-value threshold increased the number of IVs, it may have introduced additional false positives. Employing a stringent LD threshold (*r*² < 0.001) to ensure SNP independence might have excluded some effective SNPs. Excluding SNPs with a MAF≤ 0.01 improved the stability of effect estimates but may have omitted biologically relevant low-frequency variants. While removing palindromic SNPs reduced data errors, it might have overlooked critical genetic signals. Future research should focus on optimizing SNP selection criteria, exploring different *P*-value and LD thresholds, using updated reference panels, expanding sample sizes, and employing advanced techniques to manage palindromic SNPs, thereby enhancing the reliability and comprehensiveness of research findings. Finally, our study has a relatively small sample size, with only 240 cases of IC, which is indeed a limitation and may affect statistical power. Although we conducted rigorous analyses and used the largest available dataset, we recognize that the small sample size might impact the robustness of our conclusions. To address this, we employed F-statistic calculations to assess statistical power, with instrumental variables yielding results greater than 10, indicating statistical significance. Additionally, we performed sensitivity analyses, all of which support the reliability of our findings. Nevertheless, we understand that using larger datasets in future studies is essential for improving statistical power and validating our results. Despite the limited sample size, we believe our study provides a valuable foundation for exploring the potential associations between gut microbiota and interstitial cystitis, guiding future research in this field. We once again thank the reviewer for their valuable comments, and we look forward to addressing the sample size issue in future research to further enhance our contribution to this field.

Our study suggests a potential causal relationship between gut microbiota and IC based on MR analysis, biological annotation, and bulk RNA data analysis. However, these conclusions are primarily derived from predictive analysis tools and lack experimental validation. Future research should integrate *in vivo* and *in vitro* experiments to confirm the actual impact of relevant gut microbiota species and their metabolites on IC. Additionally, experimental validation is required to verify whether the key genes and bacteria identified in the analysis are genuinely involved in the pathogenesis of IC. Such validation will contribute to a more comprehensive understanding of the role of gut microbiota in IC and further substantiate the proposed mechanisms, providing a more reliable scientific foundation for the prevention and treatment of IC.

## 5 Conclusions

In this study, we employed a MR design to investigate the association between gut microbiota and IC, marking the first exploration of its kind. Through this approach, we successfully pinpointed gut microbiota linked to IC, comprising both beneficial and harmful microbial communities, thus serving as potential biomarkers. Furthermore, leveraging FUMA, we annotated SNPs to discern the genes harboring these genetic variants, indicating a potential role of gut microbiota in IC pathogenesis through gene regulation. Additionally, by comparing the expression profiles of hub genes in healthy individuals and IC patients using bulk RNA sequencing data, we potentially identified diagnostic and prognostic biomarkers associated with IC. Overall, this study offers a novel avenue for delving into the underlying pathogenesis of IC, providing insights for the development of personalized therapeutic strategies and identifying preliminary targets for future drug development.

## Data Availability

The original contributions presented in the study are included in the article/**Supplementary Material**. Further inquiries can be directed to the corresponding authors.
