# Supplementary material for: Gut microbiota and interstitial cystitis: exploring the gut-bladder axis through mendelian randomization, biological annotation and bulk RNA sequencing
Source: Front Immunol. 2024 Sep 27;15:1395580. doi: 10.3389/fimmu.2024.1395580 (PMC11466805; doi:10.3389/fimmu.2024.1395580)
Supplement: Supplementary file 1 [file DataSheet1.docx]

**Supplementary material**

**Table S2:** Self-inspection results of STROBE-MR checklist of recommended items to address in reports of Mendelian randomization studies.

**Figure S1:** Scatter plots of associations with gut microbiota (A-H, exposure) and IC (outcome) for genetic variants (used as MR instruments).

**Figure S2:** Plots for leave-one-out sensitivity analysis (MR IVW model based) for MR analysis of gut microbiota (A-H, exposure) on IC (outcome).

**Figure S3:** Funnel plots of IV precisions (1/SEIV) against the IV estimates (βIV) for the instrument variable set for analysis of gut microbiota (A-H, exposure) on IC (outcome).

**Figure S4:** The schematic of the chosen GEO datasets before to and following standardisation.

**Table S2.** Self-inspection results of STROBE-MR checklist of recommended items to address in reports of Mendelian randomization studies[1, 2]

| **Item No.** | **Section** | **Checklist item** | **Page No.** | **Relevant text from manuscript** |
| --- | --- | --- | --- | --- |
| 1 | **TITLE and ABSTRACT** | Indicate Mendelian randomization (MR) as the study’s design in the title and/or the abstract if that is a main purpose of the study | 1-2 | Causal relationship between interstitial cystitis and gut microbiota contributes to the gut-bladder axis: insights from a two-sample Mendelian randomization study, biological annotation and bulk RNA sequencing |
|  | **INTRODUCTION** |  |  |  |
| 2 | **Background** | Explain the scientific background and rationale for the reported study. What is the exposure? Is a potential causal relationship between exposure and outcome plausible? Justify why MR is a helpful method to address the study question | 2-3 | Several observational studies have indicated an association between interstitial cystitis and the composition of the gut microbiota. Gut microbiota can regulate host physiology and potentially induce disease by influencing gene expression  Associations between microbiota and IC could be bridged by relevant genes.  The main reason for its advantage in inferring causality is that MR employs the genetic variants as instrumental variables. |
| 3 | **Objectives** | State specific objectives clearly, including pre-specified causal hypotheses (if any). State that MR is a method that, under specific assumptions, intends to estimate causal effects | 4 | Based on knowledge above, we hypothesized that gut microbiome links IC and conducted a two-sample bi-directional MR analysis to elucidate the causal association between gut microbiota and IC, and further explore the potential role of several genes on these associations.  MR uses the facts that (1) genetic variants are randomly inherit one allele from each of the father and mother (namely the law of segregation assortment) and (2) alleles will be passed to offspring independently of each other (namely the law of independent assortment). Therefore, MR results are unlikely to be influenced by the environment that might confound the estimated relationship. |
|  | **METHODS** |  |  |  |
| 4 | **Study design and data sources** | Present key elements of the study design early in the article. Consider including a table listing sources of data for all phases of the study. For each data source contributing to the analysis, describe the following: |  |  |
|  | a) | Setting: Describe the study design and the underlying population, if possible. Describe the setting, locations, and relevant dates, including periods of recruitment, exposure, follow-up, and data collection, when available. | 3-4 and Table S1 | Detailed information, such as recruitment criteria of population and quality control of genetic data, can be found in the original paper (Table S1). |
|  | b) | Participants: Give the eligibility criteria, and the sources and methods of selection of participants. Report the sample size, and whether any power or sample size calculations were carried out prior to the main analysis | 3-4 and Table S1 | Detailed information, such as recruitment criteria of population and quality control of genetic data, can be found in the original paper (Table S1). |
|  | c) | Describe measurement, quality control and selection of genetic variants | 3-4 and Table S1 | Detailed information, such as recruitment criteria of population and quality control of genetic data, can be found in the original paper (Table S1). |
|  | d) | For each exposure, outcome, and other relevant variables, describe methods of assessment and diagnostic criteria for diseases | 3-4 and Table S1 | Detailed information, such as recruitment criteria of population and quality control of genetic data, can be found in the original paper (Table S1). |
|  | e) | Provide details of ethics committee approval and participant informed consent, if relevant | 3 | The data analyzed in this secondary study is publicly available from existing, published GWASs and therefore the ethical approval and informed consent have been obtained by all original studies. |
| 5 | **Assumptions** | Explicitly state the three core IV assumptions for the main analysis (relevance, independence and exclusion restriction) as well assumptions for any additional or sensitivity analysis | 4-5 | The selection of IVs, the key to ensure the accuracy and robustness of the causal inferences, must meet MR's three key assumptions.  Three key assumptions of MR: (1) genetic variants must be associated with exposures; (2) genetic variants must not be associated with confounders; (3) genetic variants must affect outcomes only through exposures, not through other pathways. |
| 6 | **Statistical methods: main analysis** | Describe statistical methods and statistics used |  |  |
|  | a) | Describe how quantitative variables were handled in the analyses (i.e., scale, units, model) | 4 | Detailed information, such as recruitment criteria of population and quality control of genetic data, can be found in the original paper (Table S1). |
|  | b) | Describe how genetic variants were handled in the analyses and, if applicable, how their weights were selected | 4 | (1) Criteria were relaxed appropriately due to the limited number of IVs at P < 5 × 10-8. Single nucleotide polymorphisms (SNPs) associated with each taxon were chosen as potential IVs at the genome-wide significance threshold (P < 1 × 10-5); (2) Using data from 1,000 samples from the European Genome Initiative as a reference panel, the linkage disequilibrium (LD) between these SNPs was calculated, and SNPs meeting an R2 < 0.001 (clumping window size = 10,000kb) were selected to reduce the likelihood of biased results; (3) SNPs with minor allele frequencies (MAF) ≤ 0.01 were excluded; and (4) An essential step in MR is to ensure that the effect of SNPs on exposure corresponds to the same allele as the effect on the outcome. |
|  | c) | Describe the MR estimator (e.g. two-stage least squares, Wald ratio) and related statistics. Detail the included covariates and, in case of two-sample MR, whether the same covariate set was used for adjustment in the two samples | 3-4 and Table S1 | The list of covariates varies between original GWASs, but always included sex and age. Details can be found in the original paper (Table S1). |
|  | d) | Explain how missing data were addressed | 3-4 and Table S1 | Detailed information, such as recruitment criteria of population and quality control of genetic data, can be found in the original paper (Table S1). |
|  | e) | If applicable, indicate how multiple testing was addressed | 5 | We applied the Bonferroni method at each taxonomic level (phylum, order, family, genus, and species) to determine distinct significance thresholds for multiple testing based on the number of bacteria at each level. The significance threshold was defined as p ≤ 0.05/n, where n represents the effective number of independent bacterial taxa at the corresponding level. |
| 7 | **Assessment of assumptions** | Describe any methods or prior knowledge used to assess the assumptions or justify their validity | 4 | F-statistics were used to estimate statistical power |
| 8 | **Sensitivity analyses and additional analyses** | Describe any sensitivity analyses or additional analyses performed (e.g. comparison of effect estimates from different approaches, independent replication, bias analytic techniques, validation of instruments, simulations) | 4,5 | Sensitivity analyses were conducted to assess the robustness of the results, including heterogeneity tests, pleiotropy tests, and leave-one-out sensitivity tests. The pleiotropy test comprised MR-Egger intercept and MR-pleiotropy residual sum and outlier (MR-PRESSO) tests. F-statistics were used to assess statistical power, Q-statistics were used to detect heterogeneity, and Bonferroni method was used for multiple comparisons |
| 9 | **Software and pre-registration** |  |  |  |
|  | a) | Name statistical software and package(s), including version and settings used | 5 | The analysis was conducted using the "TwoSampleMR (version 0.5.7)" , "MRcML", and "MRPRESSO (version 1.0)" packages within the R program (version 4.3.1).. |
|  | b) | State whether the study protocol and details were pre-registered (as well as when and where) | No Applicable | This is a secondary analysis based on summary statistics from existing, published studies. The ethical approval and informed consent have been obtained by all original studies. |
|  | **RESULTS** |  |  |  |
| 10 | **Descriptive data** |  |  |  |
|  | a) | Report the numbers of individuals at each stage of included studies and reasons for exclusion. Consider use of a flow diagram | 3-4 and Table S1 | Detailed information, such as recruitment criteria of population and quality control of genetic data, can be found in the original paper (Table S1). |
|  | b) | Report summary statistics for phenotypic exposure(s), outcome(s), and other relevant variables (e.g. means, SDs, proportions) | 3-4 and Table S1 | Summary statistics of the human gut microbiome from phylum to genus level utilized in this research were acquired from the MiBioGen consortium. This consortium conducted a large-scale, multiethnic Genome-Wide Association Study (GWAS) that integrated 16S ribosomal RNA gene sequencing and genotyping data from 18,340 participants across 24 cohorts spanning the United States, Canada, Israel, South Korea, Germany, Denmark, the Netherlands, Belgium, Sweden, Finland, and the United Kingdom...... Summary statistics for interstitial cystitis (IC) were extracted from the GWAS catalog website [3]. The study utilized the phenotype "chronic interstitial cystitis," with the GWAS summary data encompassing 456,348 European adult female participants, comprising 240 cases and 456,108 controls.  Details can be found in the original paper (Table S1). |
|  | c) | If the data sources include meta-analyses of previous studies, provide the assessments of heterogeneity across these studies | 3-4 and Table S1 | Detailed information, such as recruitment criteria of population and quality control of genetic data, can be found in the original paper (Table S1). |
|  | d) | For two-sample MR:  i.  Provide justification of the similarity of the genetic variant-exposure associations between the exposure and outcome samples  ii.  Provide information on the number of individuals who overlap between the exposure and outcome studies | 3-4 | These GWAS sample populations needed to be predominantly of European descent and largely independent of each other |
| 11 | **Main results** |  |  |  |
|  | a) | Report the associations between genetic variant and exposure, and between genetic variant and outcome, preferably on an interpretable scale | 6 | We find that eight bacterial taxa are associated with IC, namely genus *genus.Butyricimonas.id.945*, *genus.Coprococcus1.id.11301*, *order.Lactobacillales.id.1800*, *phylum.Lentisphaerae.id.2238*, *speices.Bilophila_wadsworthia*, *speices.Desulfovibrio_piger*, *speices.Oscillibacter_unclassified*, and *speices.Ruminococcus_lactaris*. The cML-MA, MR-Egger regression, weighted mode, simple mode, and weighted median methods provided comparable causal estimates to IVW in terms of magnitude and direction. |
|  | b) | Report MR estimates of the relationship between exposure and outcome, and the measures of uncertainty from the MR analysis, on an interpretable scale, such as odds ratio or relative risk per SD difference | 6 | The study revealed a positive association between IC risk and five gut microbiota: *genus.Butyricimonas.id.945* (OR= 2.273, 95% CI: 1.052-4.915, *P* = 0.037) ...... and speices.Ruminococcus_lactaris (OR= 0.273, 95% CI: 0.089-0.835, *P* = 0.023). These findings suggest a potential protective role against IC.  The limited sample size may also prevent us from providing a sufficiently precise estimate as well as 95% confidence intervals for clinical practice. |
|  | c) | If relevant, consider translating estimates of relative risk into absolute risk for a meaningful time period | No Applicable |  |
|  | d) | Consider plots to visualize results (e.g. forest plot, scatterplot of associations between genetic variants and outcome versus between genetic variants and exposure) | 6 and Figure S1 | Additional visualizations of the results, including scatter plot can be found in Figure S1. |
| 12 | **Assessment of assumptions** |  |  |  |
|  | a) | Report the assessment of the validity of the assumptions | 6-7 | The MR-Egger intercept analysis yielded non-significant results (*P* > 0.05), suggesting no genetic pleiotropy bias in the results. MR-PRESSO analysis also detected no outliers (*P* > 0.05), and Cochran's Q test found no significant heterogeneity (*P* > 0.05). Collectively, these sensitivity analyses, including Cochran's Q test, MR-Egger intercept, MR-PRESSO global test, and leave-one-out test, underscored the robustness of the MR results across both samples. |
|  | b) | Report any additional statistics (e.g., assessments of heterogeneity across genetic variants, such as *I^2^*, Q statistic or E-value) | 6-7 | The MR-Egger intercept analysis yielded non-significant results (*P* > 0.05), suggesting no genetic pleiotropy bias in the results. MR-PRESSO analysis also detected no outliers (*P* > 0.05), and Cochran's Q test found no significant heterogeneity (*P* > 0.05). Collectively, these sensitivity analyses, including Cochran's Q test, MR-Egger intercept, MR-PRESSO global test, and leave-one-out test, underscored the robustness of the MR results across both samples. |
| 13 | **Sensitivity analyses and additional analyses** |  |  |  |
|  | a) | Report any sensitivity analyses to assess the robustness of the main results to violations of the assumptions | 6-7 | The MR-Egger intercept analysis yielded non-significant results (*P* > 0.05), suggesting no genetic pleiotropy bias in the results. MR-PRESSO analysis also detected no outliers (*P* > 0.05), and Cochran's Q test found no significant heterogeneity (*P* > 0.05). Collectively, these sensitivity analyses, including Cochran's Q test, MR-Egger intercept, MR-PRESSO global test, and leave-one-out test, underscored the robustness of the MR results across both samples. |
|  | b) | Report results from other sensitivity analyses or additional analyses | 7 | Leave-one-out analysis revealed that the majority of correlated signals were not driven by a single genetic marker. |
|  | c) | Report any assessment of direction of causal relationship (e.g., bidirectional MR) | 7 | Causal effect of IC on gut microbiota  Causal effect of gut microbiota on IC |
|  | d) | When relevant, report and compare with estimates from non-MR analyses | No Applicable |  |
|  | e) | Consider additional plots to visualize results (e.g., leave-one-out analyses) | 6-7 | Additional visualizations of the results, including, funnel plots and leave-one-out plot can be found in Figure S2, S3. |
|  | **DISCUSSION** |  |  |  |
| 14 | **Key results** | Summarize key results with reference to study objectives | 8 | The investigation initiated with MR analysis of two samples to explore the potential causal link between gut microbiota and IC, employing summary statistics from GWAS. Subsequently, we delved into the molecular mechanisms through which gut microbiota influences IC development and elucidated potential host gene-microbiota associations in IC patients. To our knowledge, this represents the inaugural MR study probing the causal association between gut microbiota and IC. Our study not only holds promise for effective IC prevention and treatment strategies but also furnishes novel insights into IC pathogenesis from a gut microbiota perspective. |
| 15 | **Limitations** | Discuss limitations of the study, taking into account the validity of the IV assumptions, other sources of potential bias, and imprecision. Discuss both direction and magnitude of any potential bias and any efforts to address them | 10 | However, our study has several limitations. Firstly ...... |
| 16 | **Interpretation** |  |  |  |
|  | a) | Meaning: Give a cautious overall interpretation of results in the context of their limitations and in comparison with other studies | 8-9 | *Desulfovibrio piger*, one of the most prevalent pairs of sulfate-reducing bacteria in feces [25], releases hydrogen sulfide (H2S)…. Interestingly, we observed deleterious effects of the *Butyricimonas* and *Coprococcus* on IC, offering a novel perspective for future investigations. |
|  | b) | Mechanism: Discuss underlying biological mechanisms that could drive a potential causal relationship between the investigated exposure and the outcome, and whether the gene-environment equivalence assumption is reasonable. Use causal language carefully, clarifying that IV estimates may provide causal effects only under certain assumptions | 9 | Our findings suggest that gut microbiota with beneficial roles may impact channel regulator activity, protein binding, cadherin binding, and other pathways.... Therefore, SPTBN1, PSME4, CHAC2, ERLEC1, ASB3, STAT5A, and STAT3 could potentially represent novel genes implicated in the underlying pathogenesis of IC and serve as therapeutic targets. |
|  | c) | Clinical relevance: Discuss whether the results have clinical or public policy relevance, and to what extent they inform effect sizes of possible interventions | 9-10 | Spectrin Beta, Non-Erythrocytic 1 (SPTBN1) is a protein-coding gene known for its role in maintaining neuronal morphological stability, cellular structure, and cell signaling. Additionally, it contributes to synapse formation and normal neuronal function…. Hence, we hypothesize that gut microbiota may contribute to the onset and progression of IC inflammation by modulating the JAK/STAT signaling pathway, thereby influencing IC inflammation development. In conclusion, further studies are warranted to elucidate the mechanisms underlying the action of these genes in IC and their therapeutic potential. |
| 17 | **Generalizability** | Discuss the generalizability of the study results (a) to other populations, (b) across other exposure periods/timings, and (c) across other levels of exposure | 10 | Firstly, the majority of patients in the GWAS summary data used were of European descent, with limited representation from other ethnicities. This imbalance may introduce estimation bias and limit the generalizability of our findings. Consequently, the results may not be fully applicable to individuals of non-European origin. Future MR studies exploring the causal relationship between gut microbiota and IC should include diverse European and non-European populations to enhance generalizability. Secondly, subgroup analyses, such as with Hunner-type and non-Hunner-type, were not possible since pooled data, rather than raw data, were used in the analyses, whereas symptoms are usually more severe in the former [61]. Moreover, IC predominantly affects the female population, and gender-specific differences exist in gut microbiota composition[ 62]. Unfortunately, our study did not conduct separate analyses for both genders. Future investigations should incorporate gender-specific MR analysis to provide comprehensive insights into the relationship between gut microbiota and IC across different populations. |
|  | **OTHER INFORMATION** |  |  |  |
| 18 | **Funding** | Describe sources of funding and the role of funders in the present study and, if applicable, sources of funding for the databases and original study or studies on which the present study is based | 11 | Funding Sources |
| 19 | **Data and data sharing** | Provide the data used to perform all analyses or report where and how the data can be accessed, and reference these sources in the article. Provide the statistical code needed to reproduce the results in the article, or report whether the code is publicly accessible and if so, where | 11 | Availability of data and materials |
| 20 | **Conflicts of Interest** | All authors should declare all potential conflicts of interest | 10 | The authors declare that they have no conflict of interest. |

This checklist is copyrighted by the Equator Network under the Creative Commons Attribution 3.0 Unported (CC BY 3.0) license.

1. Skrivankova VW, Richmond RC, Woolf BAR, Yarmolinsky J, Davies NM, Swanson SA, et al. Strengthening the Reporting of Observational Studies in Epidemiology Using Mendelian Randomization: The STROBE-MR Statement. JAMA. 2021;326:1614.

2. Skrivankova VW, Richmond RC, Woolf BAR, Davies NM, Swanson SA, VanderWeele TJ, et al. Strengthening the reporting of observational studies in epidemiology using mendelian randomisation (STROBE-MR): explanation and elaboration. BMJ. 2021;:n2233.


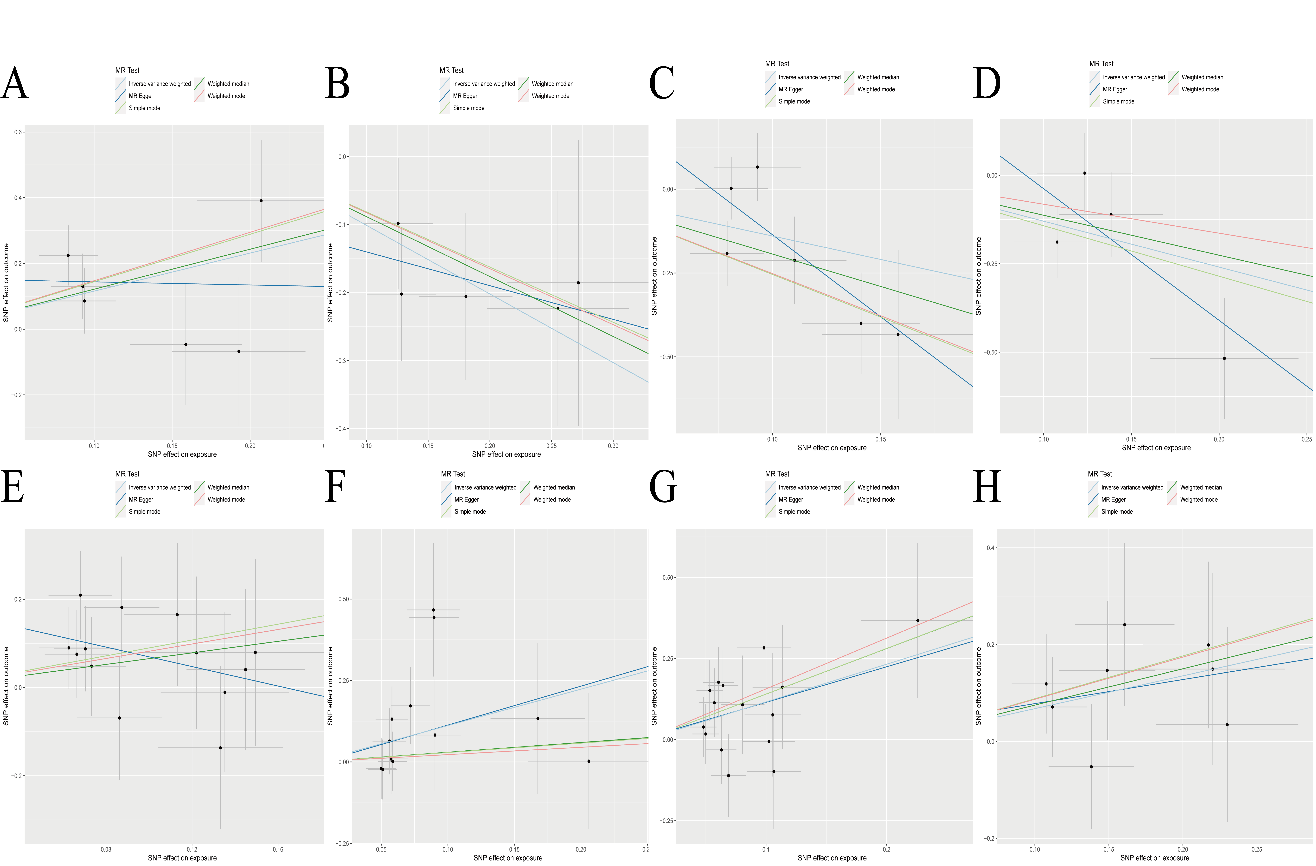


**Figure S1.** **Scatter plots of associations with gut microbiota (A-H, exposure) and IC (outcome) for genetic variants (used as MR instruments)**

**(A)** *speices.Bilophila_wadsworthia*; **(B)** *speices.Desulfovibrio_piger*; **(C)** *speices.Oscillibacter_unclassified*; **(D)** *speices.Ruminococcus_lactaris*; **(E)** *genus.Butyricimonas.id.945; (F) genus.Coprococcus1.id.11301*; **(G)** *order.Lactobacillales.id.1800*; **(H)** *phylum.Lentisphaerae.id.2238*. The gray line represents 95% confidence intervals. The light blue line shows the result of standard MR analysis (IVW), dark blue line for MR Egger, light green line for Simple mode, dark green line for Weighted median, and red line for Weighted mode).


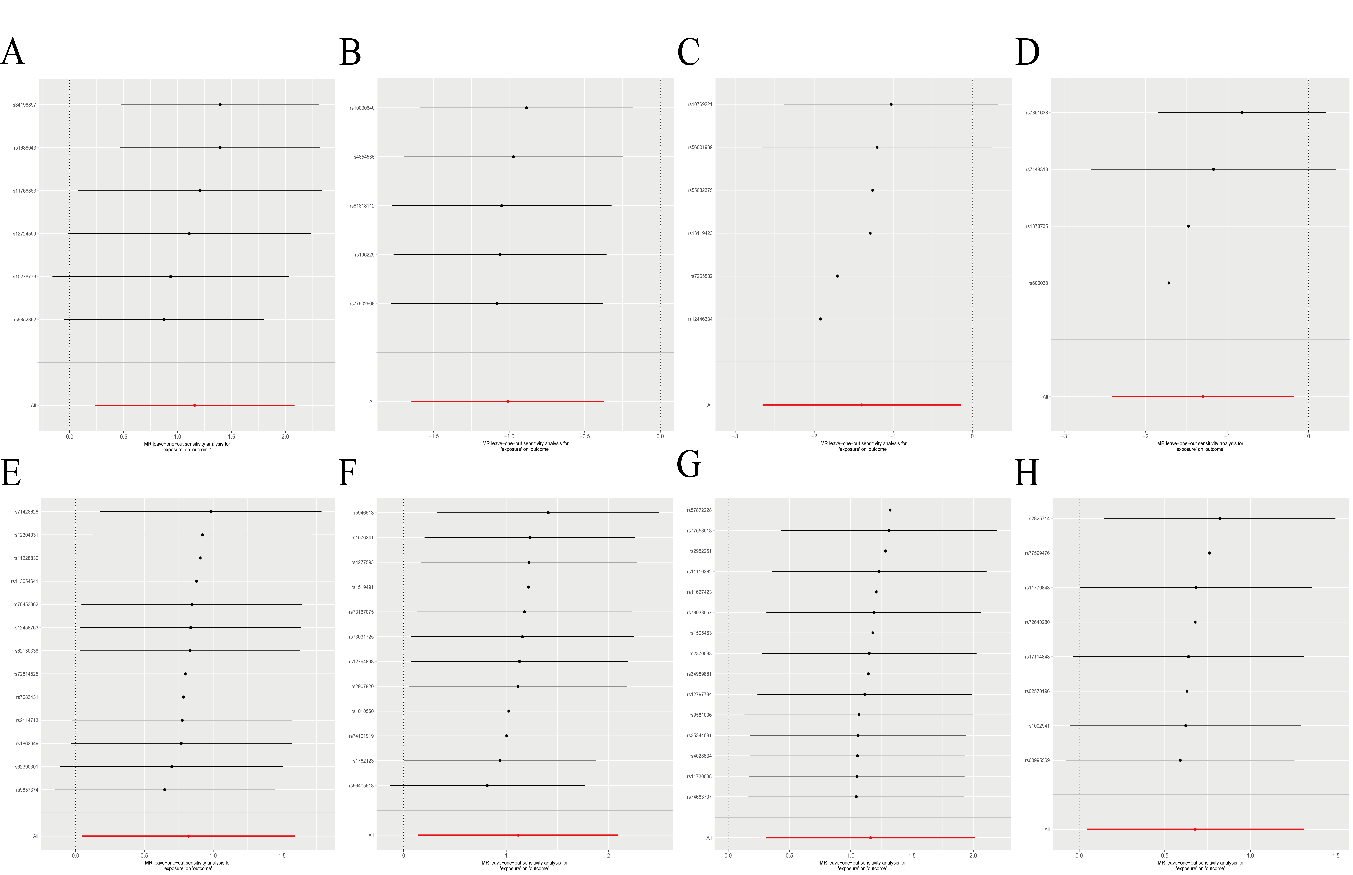


**Figure S2.** **Plots for leave-one-out sensitivity analysis (MR IVW model based) for MR analysis of gut microbiota (A-H, exposure) on IC (outcome).**

**(A)** *speices.Bilophila_wadsworthia*; **(B)** *speices.Desulfovibrio_piger*; **(C)** *speices.Oscillibacter_unclassified*; **(D)** *speices.Ruminococcus_lactaris*; **(E)** *genus.Butyricimonas.id.945; (F) genus.Coprococcus1.id.11301*; **(G)** *order.Lactobacillales.id.1800*; **(H)** *phylum.Lentisphaerae.id.2238*. The leave-one-out analysis showed none of the individual genetic markers are driving the majority of the association signal. The solid lines represent 95% confidence intervals.

**
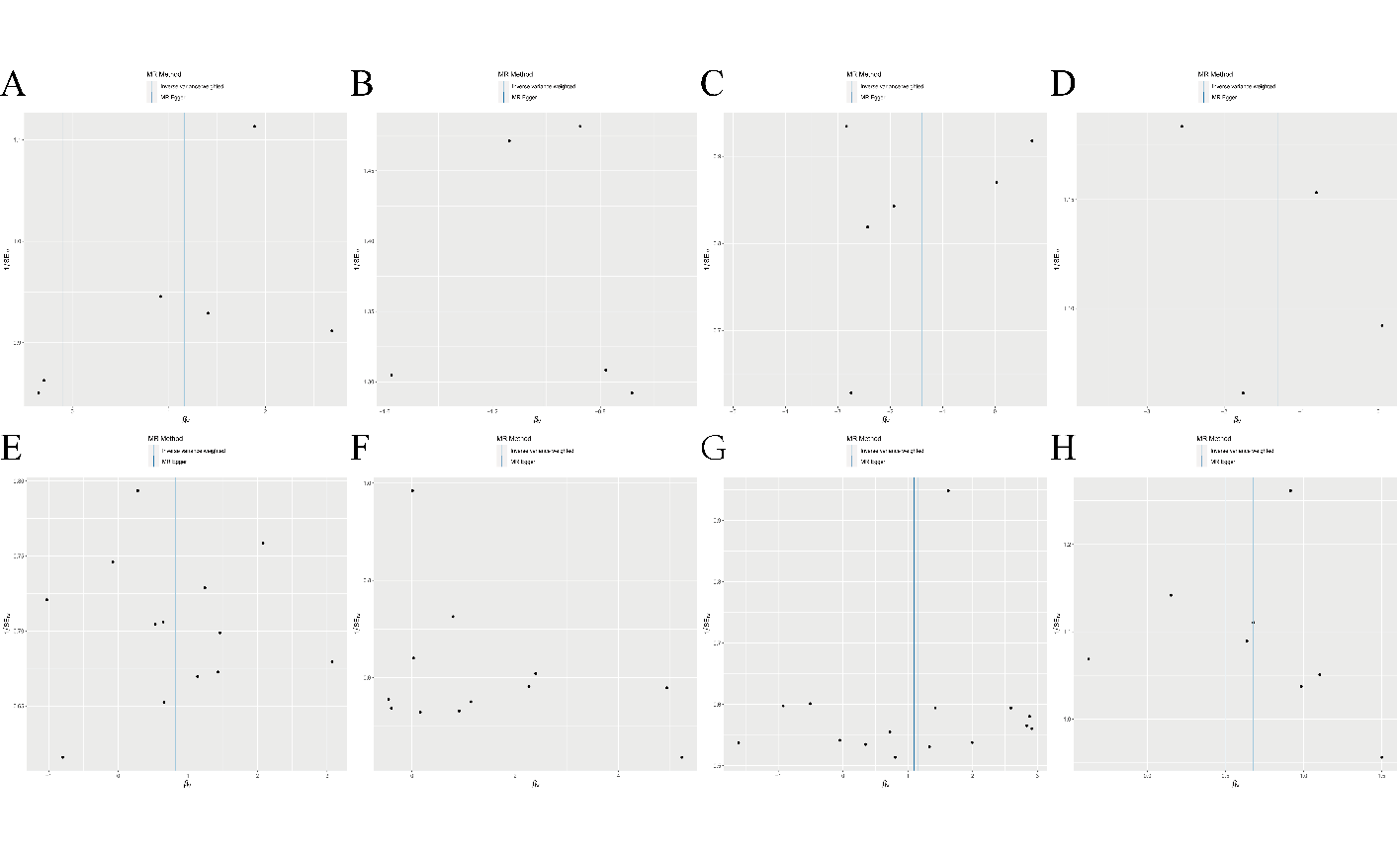
**

**Figure S3. Funnel plots of IV precisions (1/SE_IV_) against the IV estimates (β_IV_) for the instrument variable set for analysis of gut microbiota (A-H, exposure) on IC (outcome).**

1. *speices.Bilophila_wadsworthia*; **(B)** *speices.Desulfovibrio_piger*; **(C)** *speices.Oscillibacter_unclassified*; **(D)** *speices.Ruminococcus_lactaris*; **(E)** *genus.Butyricimonas.id.945; (F) genus.Coprococcus1.id.11301*; **(G)** *order.Lactobacillales.id.1800*; **(H)** *phylum.Lentisphaerae.id.2238*. The solid lines illustrate estimates of the causal effect, and different MR methods are indicated with different colors (light blue for IVW and dark blue for Egger). IV, instrument variable.

**
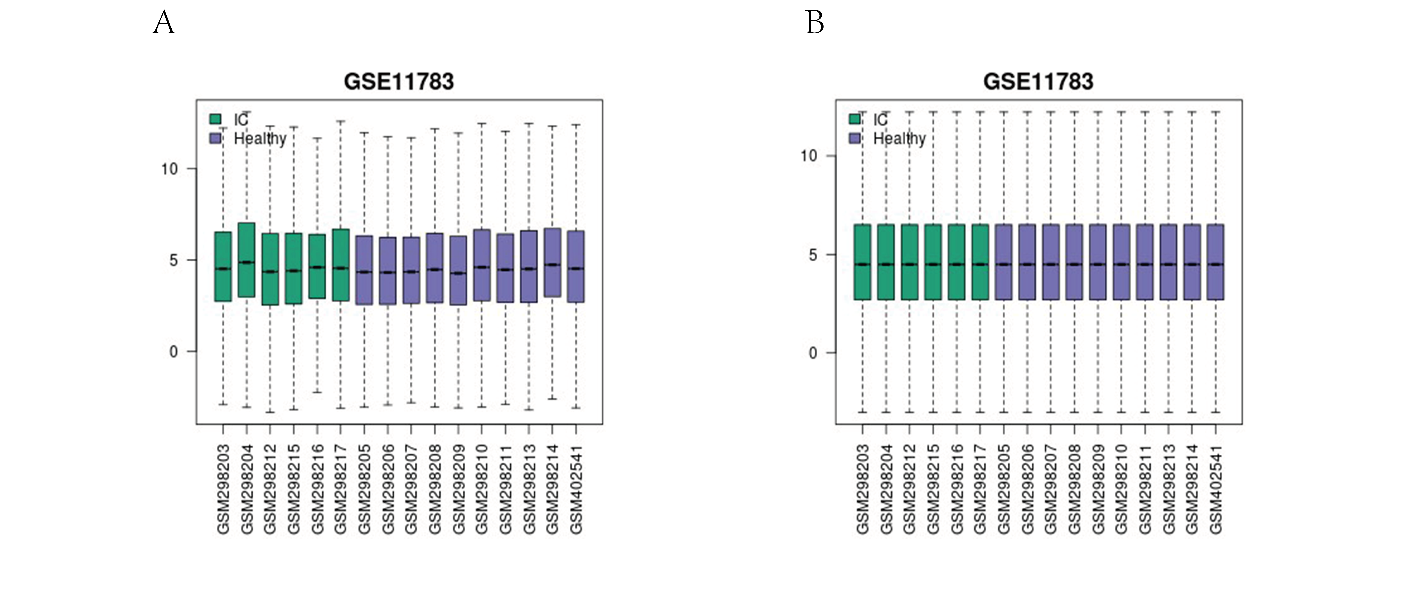
**

**Figure S4: The schematic of the chosen GEO datasets before to and following standardisation. (A)** The GSE11783 dataset was not standardised. (B) The GSE11783 dataset has undergone standardisation.
